# Economic Freedom: The Top, the Bottom, and the Reality. I. 1997–2007

**DOI:** 10.3390/e24010038

**Published:** 2021-12-25

**Authors:** Marcel Ausloos, Philippe Bronlet

**Affiliations:** 1 School of Business, University of Leicester, Brookfield, Leicester LE2 1RQ, UK; 2 Department of Statistics and Econometrics, Bucharest University of Economic Studies, 6 Piata Romana, 1st District, 010374 Bucharest, Romania; 3 Group of Researchers for Applications of Physics in Economy and Sociology (GRAPES), Sart Tilman, B-4031 Liege, Belgium; p.bronlet@gmail.com; 4 Advanced Mechanical and Optical Systems (AMOS), Rue des Chasseurs Ardennais 2, B-4031 Liege, Belgium

**Keywords:** Economic Freedom of the World index, Index of Economic Freedom, rank-size law technique, power law behaviour, exponential behaviour

## Abstract

We recall the historically admitted prerequisites of Economic Freedom (EF). We have examined 908 data points for the Economic Freedom of the World (EFW) index and 1884 points for the Index of Economic Freedom (IEF); the studied periods are 2000–2006 and 1997–2007, respectively, thereby following the Berlin wall collapse, and including 11 September 2001. After discussing EFW index and IEF, in order to compare the indices, one needs to study their overlap in time and space. That leaves 138 countries to be examined over a period extending from 2000 to 2006, thus 2 sets of 862 data points. The data analysis pertains to the rank-size law technique. It is examined whether the distributions obey an exponential or a power law. A correlation with the country’s Gross Domestic Product (GDP), an admittedly major determinant of EF, follows, distinguishing regional aspects, i.e., defining 6 continents. Semi-log plots show that the EFW-rank relationship is exponential for countries of high rank (≥20); overall the log–log plots point to a behaviour close to a power law. In contrast, for the IEF, the overall ranking has an exponential behaviour; but the log–log plots point to the existence of a transitional point between two different power laws, i.e., near rank 10. Moreover, log–log plots of the EFW index relationship to country GDP are characterised by a power law, with a rather stable exponent (γ≃0.674) as a function of time. In contrast, log–log plots of the IEF relationship with the country’s gross domestic product point to a downward evolutive power law as a function of time. Markedly the two studied indices provide different aspects of EF.

## 1. Introduction

Numerous empirical studies [1] pretend to show that Economic Freedom (EF) favours economic growth, prosperity, poverty reduction, and has many other beneficial effects, beside being also a necessary condition for the development of democracy. However, before proposing modern theories of Economic Freedom, it seems that one should first wonder about the EF definition, and have proofs that Economic Freedom exists. The goal of this paper is to study the world EF situation before the recent (21st century) economic crisis. A second paper is intended for later years as explained below. In brief, this is due to different definitions and changes in geo-political economic conditions. It is expected that the paper can be useful for econo-physicists and other researchers, due to the somewhat original approach, more numerical, i.e., along the lines of econophysics thought.

The oldest of these publications, *The Wealth of Nations* by Adam Smith in 1776, shows that the preservation of individual freedom to pursue their own interests is due to the necessity of creating a social and more prosperous civilisation [2]. On the other hand, protectionism and trade performed under a monopoly (like that of the British empire at the time of Adam Smith) serves the purpose of preserving the status quo and privileging a handful of elites. Frederic Bastiat shows, in *Economics Harmonies* [3], that all human actions lead to care and harmony if these actions are motivated by private considerations. Thus, Bastiat recommends, or even advocates, “liberty” [4], in our own words, EF contains so much creativity that it leads to many opportunities for bettering human life.

But what is “Economic Freedom” (EF)? A simple definition among many similarly proposed by others may be as follows: The freedom of the economy is the freedom to produce, exchange and consume any goods and services acquired without use force, fraud, or theft.

In order to have a more complete appraisal of EF, one might consider James Gwartney and Robert Lawson’s article [5]. Gwartney and Lawson do not give a proper term for economic freedom, but claim to provide all the conditions to be met in order to obtain “economic freedom”: in brief, the foundations of any “economic freedom” is respect for the “rule of law”, of property and privacy, i.e., “right to own”, and demands freedom for agents wishing to enter into contracts, i.e., “freedom to contract”. Thus, before, measuring EF and discussing such measures, let us briefly examine the framework in the following three subsections.

### 1.1. Rule of Law

Many theoreticians of economic liberalism maintain that the aim of the prerequisites for EF is the establishment of a rule of law; e.g., [6]. A “rule of law” (*“Etat de droit”*) is an institutional system in which the government and the individuals are subject to the law. This right shall apply in an identical way to each individual and to all economic agents.

This principle of equality of individuals before the law is the guarantee that the fundamental rights of citizens will not be violated by those in power. It also excludes any form of privilege, i.e., the application of the law with the purpose of favouring one group of people over another. It restricts also any arbitrary application of the law. Otherwise, one of these “misactions“ would lead to a restriction of economic freedom.

### 1.2. The Right to Own

The second prerequisite for EF is the respect of the individual rights to own property. To achieve this, a system must be established which ensures the right to use (usus) and to profit (fructus) from this property. The system shall also ensure the right to transfer this property to another person as long as they are both consenting.

These fundamental rights are the guarantees that individuals will be able to be autonomous and will have the opportunity to seek to achieve their own goals. Many economists, such as Milton Friedman [7,8] or Murray Rothbard [9,10], consider the right of ownership as the most fundamental of the rights, of all other rights. It guarantees individuals to have individual freedom and allows for better personal development than otherwise, under a regime of coercion. It also reduces uncertainty and encourages investment by creating favourable conditions for economic development.

Empirical studies [11] show that countries with a right to own have an economic growth rate almost twice larger than countries where this right is not respected. According to (the Peruvian economist) Hernando de Soto [12], a large part of the poverty in third world countries is caused by the system’s lack of favouring some equality and by the absence of a right of ownership.

### 1.3. Freedom to Contract

A contract is an agreement between two or more parties, having the purpose of establishing obligations at the expense of each of those parts. The freedom to contract contains therefore the right to choose the parties with which the contract is formed and to agree on the content of this contract (what to give, to do, or not to do). The parties have the right to choose the subject of the contract, but once the contract has been made, they are obliged to fulfil the terms of the contract.

The main economic function of contracts is to transfer rights of one individual’s property to another person.

### 1.4. Other Definitions of Economic Freedom

The Gwartney and Lawson definition [5] is an ideal one, but accepted by classic liberal economists. It is intimately linked to a respect for the law which in so doing protects individuals against external aggression that would aim to take ownership of their property. This definition is valid only in a “non-negative legal context”.

There are many other definitions of EF but none is unanimously accepted. Examples of “economic freedom” in a “positive law context” are given by Amartya Sen [13]; Amartya Sen argues for an understanding of freedom in terms of capacity of an individual to achieve his/her own goals. Notice that in a similar line of thought, Goodin, Rice, Parpo, and Eriksson [14] propose to measure “freedom”, even outside financial or economic considerations, from the available time that people have in participating in an activity so chosen by them.

### 1.5. Paper Content

However, before a theory of economic freedom is proposed, should one not first have proofs of where and when economic freedom exists? In fact, these questions demand a study of other highly fundamental research questions, in particular about the measurement(s) of economic freedom(s?) themselves, and on the meaning of the measures (so called “indices”). Immediately tied to the former and the latter, the correlations with other socio-economic measures should be considered in order to provide stylised data for some preparation of modelling, later on with determinants or/and components. These are huge challenges having led to a vast literature.

Thus, even though the literature is enormous, on many aspects, we have only considered some, in our opinion, very elementary but fundamental, ground level basis, accepting two types of measures, explicitly defined in Section 2: the Economic Freedom of the World (EFW) index and the Index of Economic Freedom (IEF). We have examined 908 data points for the EFW index and 1884 points for the IEF; the studied periods cover 2000–2006 and 1997–2007, respectively, thereby following the 9 November 1989 Berlin wall collapse and including 11 September 2001. Notice that we presently exclude the 2008 financial crisis, and the following years, due to recent economic, geopolitical, changes, and because a new definition of the IEF was recently implemented. Some further work is intended over the more recent period (to be paper II.) in order to provide a complementary analysis. Paper II will also contrast the findings, whence prompting any dynamic aspect.

In order to compare the indices, one needs to study their overlap in time and space. That leaves 138 countries to be examined over a period extending from 2000 to 2006, thus 2 sets of 862 data points. Since each country presents a combination of freedoms, and restrictions to freedoms, it is of interest to observe whether the country ranking contains or hides such a variety of dimensions. Due to the aimed scope of this paper, we will only consider the most often admitted primary determinant of a country’s economic growth (EG), i.e., the country’s Gross Domestic Product (GDP).

Thus, our data analysis pertains to the rank-size law technique. It is going to be examined whether the measures of EF have a statistical distribution which follows either an exponential or a power law. This is a sort of research question not considered in the classical realms of economics, but should be of interest in econophysics. A correlation with the country’s gross domestic product (GDP) follows, distinguishing regional aspects, i.e., defining 6 continents.

The table of contents of this paper may be as follows:

In Section 2, we recall the definition and content of the Economic Freedom of the World (EFW) Index and the Index of Economic Freedom (IEF), respectively.

In Section 3, we present the extracted data, i.e., 908 data points for the EFW index and 1883 for the IEF on the studied periods, 2000–2006 and 1997–2007, respectively.

In Section 4, we provide the empirical laws, on one hand, the rank-size laws for both indices, plus, on the other hand, the (regression) relationship between such indices and the gross domestic product of the countries of interest. We also provide a study of regional aspects through a grouping of countries according to their geographic positions.

In Section 5, we provide conclusions pointing to the weak evolution of indices over the considered time interval. We suggest lines for further research.

## 2. Economic Freedom Indices

We position our paper within the scholarly contributions having investigated, on one hand “measures of Economic Freedom” in modern times, and the link between EF and EG. Our article explores this possibility by means of a regional analysis, which we conduct on two indicators. Let us summarise the literature from such points of view.

### 2.1. Economic Measures

#### 2.1.1. Economic Freedom of the World (EFW) Index

The Economic Freedom of the World (EFW) Index, published by the Fraser Institute [15], is the result of a project spanning 20 years. It was developed after a set of conferences given by Milton Friedman and Michael Walker between 1986 and 1994, in a project gathering more than 60 of the greatest economists of the time [16]. The aim was to create a (“strong”) base with quantifiable and objective data following a transparent procedure. Thus, anyone could use the index, whatever their goals and political ideals.

The EFW index measures the degree of economic freedom in 5 major “areas”:

- The size of the government, i.e., public expenditure, taxes, influence on the economy
- The legal structure which guarantees the right to own
- The access to a healthy currency
- Freedom in international trade
- Regulation of costs, work and economy

For each of these 5 domains, several variables are measured, resulting in a set of 21 components included in the index. Each component is placed on a scale going from 0 to 10. The value 0 refers to zero freedom while the value 10 represents total freedom. Once these components are quantified, they are averaged in order to obtain the index value.

Several methods have been studied for doing such an average: without being exhaustive, one considers the weight equivalent to each component; another gives an inversely proportional weight to the standard error of the distribution of the component in the various studied countries. A third method calls upon a panel of economists who estimate the weight that each component must have; the final weight being the average weight obtained from the panel members’ appraisals. A fourth method uses the primary component analysis technique to determine each weight. This latter method has the advantage of reducing the importance of anomalies (outliers) in estimating correlations between the components.

Since none of these methods is really satisfactory (from our investigations, the index does not seem to be very sensitive to changes in weight), the weight choice is not further discussed, and taken as the most simple one. Thus, an equal weight for each component is chosen in the forthcoming analysis here below. The index, so constructed, provides a value between 0 and 10 for each country. A country with an index value close to 10 is a country where “economic freedom” is “very large”. A country with a value close to 0 is a country where EF is “non-existent”.

Of course, it is expected that each country presents a “combination of freedoms”. Recently, Lawson et al. [17] have reviewed the determinants of EF, with a time dependent point of view. Some of the most consistent findings are that current levels of EF are strongly correlated with past levels. Lawson et al. deduce that freer countries have more difficulty continuing to improve their economic freedom.

#### 2.1.2. Index of Economic Freedom (IEF)

Another measure of economic freedom, published by the Heritage Foundation [18] and the Wall Street Journal [19], is the Index of Economic Freedom (IEF), which was initiated in 1995 [20].

The index was built on a set of 10 specific components [21]:

- Tax freedom: measures the importance of fiscal fees imposed by the government on the income of individuals and businesses.
- Government spending: measures the total government spending.
- Free trade: it measures the absence of commercial barriers, affecting the import and export of goods or services.
- Investment freedom: measures the freedom of capital flows.
- Financial freedom: measurement of the independence from the government of credit and banking systems.
- Property rights: they are measures of the ease with which individuals acquire a property of their own.
- Corruption: measures the importance of corruption in the economic world.
- Business undertaking freedom: measures the ease with which it is possible to create, develop and close a business.
- Monetary freedom: measures price stability in relation to a price control.
- Labor Code (This item has been added in 2007. Moreover, in 2017, the Heritage Foundation made some methodological changes; the IEF has 12 components nowadays. The new components are “Judicial Effectiveness” belonging to the Rule of Law pillar and “Fiscal Health” as the new factor of the Government size pillar.): it measures the ease with which workers and companies interact without restriction from the state government.

Some of these components are the results of an assembly of additional measures. Each of these components is measured on a scale of 0 to 100. The value 100 represents the maximum freedom. The index was obtained in averaging these 10 components (with an equal weight for each of them).

Notice that more recently, Dialga and Vallée [22] dealt with “methodological issues in the Index of Economic Freedom”, indicating that two components, “1. Tax Freedom” and “Government Spending”, which define the “2. Government Size” pillar, are negatively correlated to the other “pillars”, whence making the index very unstable and thus impairing the country ranking.

### 2.2. Economic Growth

Most empirical studies, e.g., [23,24,25,26,27] provide evidence that economic freedom, as measured by the Economic Freedom of the World Index, is related to economic growth, income, standard of living, low corruption, etc. Much evidence shows that economic freedom leads to economic growth even where countries have limited political freedom [28,29,30]. The reverse is not true. The case of IEF is less studied [31]. In most cases, the question turns upon the level of importance of the various independent variables.

One of the first papers that explored the relationship between EF and growth was by Islam [32]. The first study concerning the analysis of the link between different components of EF and economic growth seems due to Ayal and Karras [25]. However identifying which aspects of EF are more conducive to growth has proven difficult, due to multicollinearity among the index areas [33]. Due to the more basic aim of our paper, we will not discuss any further regression models nor (Granger) causality in the freedom–growth relationship, here, whence reducing to a somewhat limited literature review. Nevertheless, for some completeness, let us point out a few papers, either considering EF–EG from the EFW [34,35] or the IEF [31] point of view.

### 2.3. Criticism/Limitations

These types of indices are often criticised for their methodology. Some “economists” criticise the economic basis on which such indices are based. They consider the measures to be too restrictive and demand that they should include a broader range of freedom concepts. Others, such as John Miller [36], argue that the relationship found for example between a high life level and such indices is the biased result of choices made in the construction of some index. Others, like Heckelman and Stroup [37], criticise the method used in order to average components, which they consider to be arbitrary. See also the previous mention of Dialga and Vallée’s recent finding [22].

## 3. Data

In order to study the spread of EF around the world, its evolution during this past decade, and subsequently its impact on the richness of the world, it is necessary to obtain the values of the EFW index and of the IEF together with the gross domestic product for the studied countries.

The EFW index values, obtained from the portal www.freetheworld.com, accessed on 30 October 2006 [38], are provided for 140 countries in the 2000–2006 period, i.e., over 7 years. The values of the IEF can be found on the site of the “Heritage Foundation” [39]. The indices are given for 157 countries in the (12 years) period 1997–2007. The values of the Gross Domestic Product per capita (GDP) of countries for corresponding periods may be downloaded from the IMF website [40]. All values are annual data.

We point out that it was unfortunately necessary to exclude certain countries for which the data was unavailable for various reasons. This is, for example, the case of Iraq. Iraq’s second war has made the measurement of economic indicators quite dubious: the values obtained for the IEF and EFW indices or for GDP could not be considered to be significant. That being said, there are still 908 data points for the EFW index and 1784 for the IEF for the studied periods.

### 3.1. Statistical Characteristics of Indices Distribution

**Figure 1:**
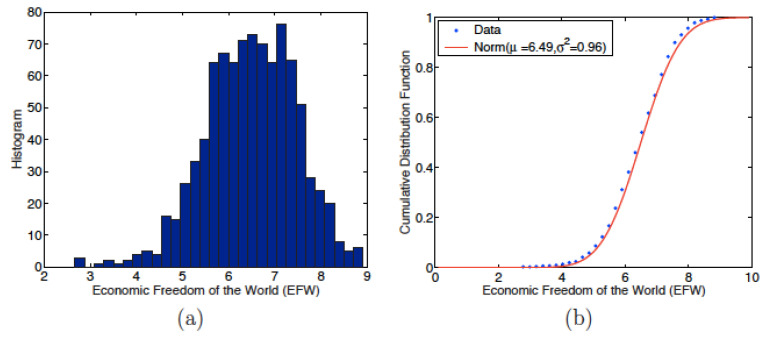
(**a**) Economic Freedom of the World (EFW) histogram for 908 data points, i.e., when available for all (140) countries and for all (7) years; (**b**) cumulative probability density for the EFW and normal distribution fit with mean μ = 6.49 and variance $\sigma^{2}$ = 0.96.

**Figure 2:**
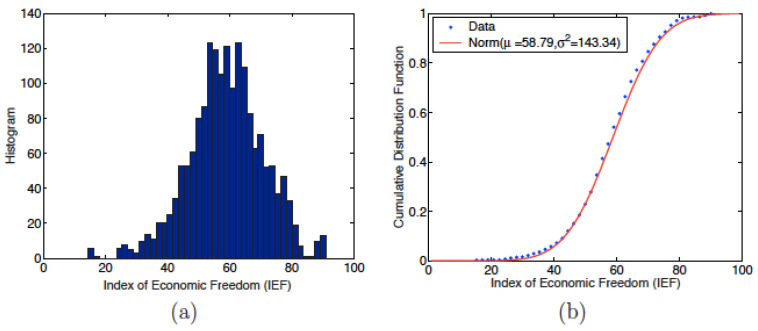
(**a**) Index of Economic Freedom (IEF) histogram for 1784 data points; (**b**) cumulative probability density for the IEF and normal distribution fit with mean μ = 58.79 and variance $\sigma^{2}$ = 143.34.

The first step in the study of the indices concerns the distribution of their values. The histograms and cumulated probability densities of the EFW and of the IEF are reproduced in Figure 1 and Figure 2, respectively. The main statistical characteristics (mean, standard deviation, variance, coefficient of variation, skewness and kurtosis) of these distributions are included in Table 1.

Figure 1 and Figure 2 suggest that both indices follow a normal law slightly displaced to the right, i.e., to values greater than the median values, whence the negative skewness. This impression is reinforced by the average values of the indices: 6.49 for the EFW index and 58.79 for IEF, see Table 1. These two averages are greater than the corresponding median values: 5 in the case of EFW and 50 in the IEF. The skewness is negative for both indices: −0.3567 for the EFW index and −0.2373 for the IEF, see Table 1, confirming that the probability densities are no longer important for values above the median. These features show that the economies of the studied countries are generally more free than constrained.

In order to confirm that the distributions follow a normal law, a Kolmogorov–Smirnov (KS) test is performed. The results of the tests are shown in Table 2. The KS distances, DKS = 0.0399 for EFW and 0.0310 for IEF, are lower than the “critical values“ of the normal distribution, 0.0449 for EFW and 0.0321 for the IEF. In addition, *p*-values, 0.1088 for the EFW index and 0.0633 for the IEF, are above the 5% significance level; thus the KS tests are considered to lead to statistically significant features. It is therefore possible to conclude that the EFW index and IEF values follow a normal law with μ = 6.49 and 58.79 and variance $\sigma^{2}$ = 0.96 and 143.34 respectively, i.e., the standard distribution (SD) is equal to 0.98 and 11.98, respectively.

### 3.2. EFW Index in Year 2006

For example, consider a specific year, 2006. Table 3 shows the EFW index values for the 20 freest countries for the year 2006. Hong Kong, Singapore, and New Zealand occupy the first 3 places. The rest of the top 20 is made up of the great Anglo-Saxon countries (USA, Canada, Australia) and European countries (Switzerland, United Kingdom, Ireland, Estonia, Iceland, Denmark, Finland, Austria, Netherlands, Germany, Slovakia). It should be noted that there is one South American country, Chile (in 6th position) and one country from the Arabian Peninsula, Kuwait (in 19-th position).

In constrast, Table 4 shows the EFW index for the 21 least free countries in 2006. It is remarkable that the least free countries are mainly grouped in Africa: 16 out of the 21 last countries.

### 3.3. IEF in Year 2006

Similarly, Table 5 and Table 6 list the IEF values for the 20 freest countries and the 20 least free countries, respectively. The former British colonies still dominate the ranking. Hong Kong and Singapore occupy the top 2 places in the ranking. The big Anglo-Saxon (United States, United Kingdom, Australia, and Canada) countries are also in the top 20. Among all the regions of the world, Europe has the largest number of countries in the top 20 (9 of the 20 countries are European).

As in the case of the EFW index, a large majority of the “less free” countries are in Africa (10 out of 20 countries). The (last) Communist Countries (North Korea and Cuba) are appearing in the 2 last places of the ranking.

### 3.4. Regional Evolution of Economic Freedom

In order to study the geographical distribution of economic freedom, it is possible to calculate an “average freedom value” for the six major continents (Africa, Asia, Europe, North America, Oceania, and South America). The distribution of countries by continent is carried out by following the geographical scheme of the United Nations Statistics Division [41]. This partition has been chosen because it has been developed with the aim of conducting statistical studies relevant to the various regions. However, the calculation of such an average selected is not a simple arithmetic mean. It does not make sense to give a similar weight to the United States and e.g., to Ecuador, to China, or to Vietnam. Instead, we consider that the weight should depend on the country’s contribution to the world economy, for example through the GDP. Thereafter, the weight is given by

(1) $$w_{i}=\frac{GDP_{i}}{\sum_{j=1}^{N}GDP_{j}}$$

where $w_{i}$ represents the weight of the country *i* and $GDP_{j}$, the internal product country *j*.

**Figure 3:**
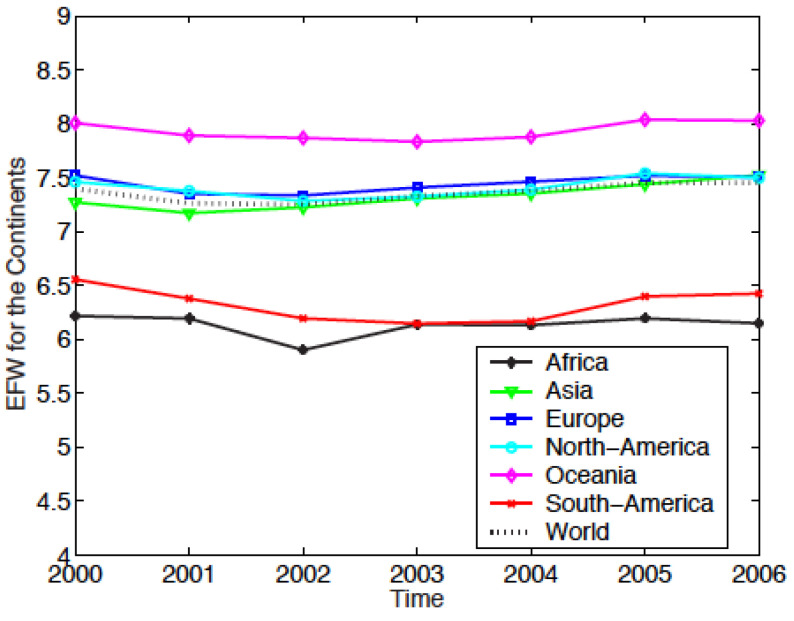
Yearly evolution of the Economic Freedom of the World (EFW) Index for the six continents (Africa, Asia, Europe, North America, Oceania, and South America). The index calculation for a region results from a weighted averaging of the indices of the countries belonging to the specific region. The weight of a country is the ratio of the GDP of the country to the GDP of the world economy.

**Figure 4:**
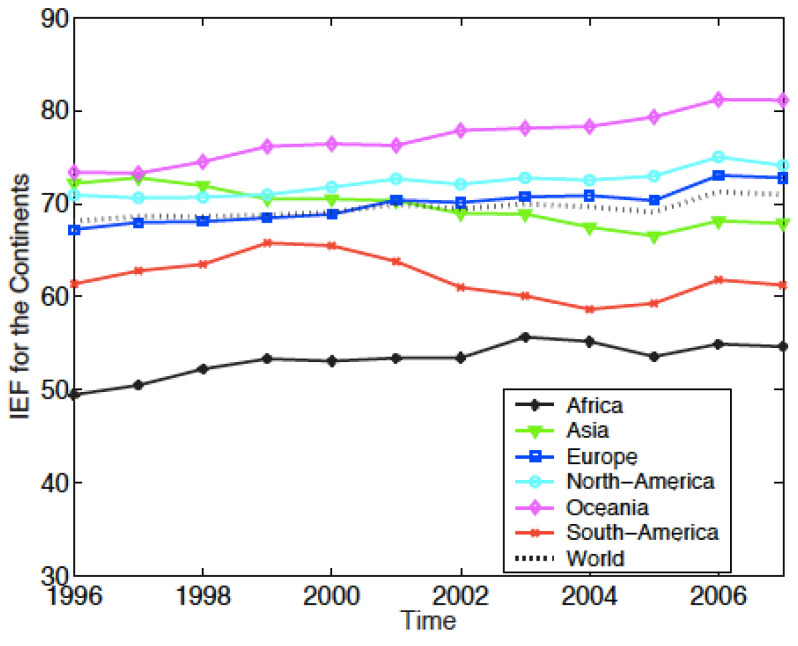
Yearly evolution of the Index of Economic Freedom (IEF) for the six continents (Africa, Asia, Europe, North America, Oceania, and South America). The index calculation for a region results from a weighted averaging of the indices of the countries belonging to the specific region. The weight of a country is in the ratio of the GDP of the country to the GDP of the world economy.

The evolutions of the EF for the 6 continents, obtained by this method are reproduced in Figure 3 for the EFW index, and in Figure 4 for the IEF.

For the EFW index, Figure 3 shows that Oceania is the the freest of the six regions, with an index value ≃8, relatively stable of the 7 years. Europe, North America, and Asia are *ex aequo* with a value ≃7.5, which represents the world average value. Africa is the less free region and South America does not fare much better.

For the IEF, Figure 4 also shows that Oceania is the freest region with an ever increasing value. It goes from 73.36 in 1996 to 81 in 2007. Europe and North America follow the same evolution and have almost identical values. Asia regresses in terms of “economic freedom”, even though there is a slight improvement in the last two years. It goes from 72.2 to 67.9 with a minimum value equal to 66.4 in 2005. Africa is again the least free region of the world, but progresses over the 12 years period. Overall, the world average freedom is rising from 68 in 1996 to 71 in 2007.

The “rate changes” appear to be different from one index to the other; this is due to the periods of study. Indeed, if the study period is restricted to 2000–2006 for the IEF, the results so obtained for both indices are almost identical. The slight differences are explained by the fact that the IEF is “more conservative” than the EFW; the IEF leads to values lower than EFW for a country. This topic is discussed further in Section 3.6.

### 3.5. Exponential Versus Power Law Behaviour

In this section, countries are ranked according to the value of the indices in a conventional order: a low ranking indicates that the country belongs to the group of the freest countries in the world. Conversely, a “high” rank means that the country has an index value, whence a low EF as compared to others.

The goal here is to determine, the so called “rank-size” law, once the countries are ranked, in particular whether the indices follow an exponential or a power law (These are the two most simple analytical functions carried over from statistical physics to econophysics; whence their mathematical origin is well known and not further discussed.), i.e.,

(2) $$INDEX∼e^{\lambda r}$$

or

(3) $$INDEX∼r^{\nu }$$

where *r* is the rank of the country; λ and ν are characteristic exponents. The latter equation corresponds to the (so called Zipf) rank-size law [42], if ν=−1.

**Figure 5:**
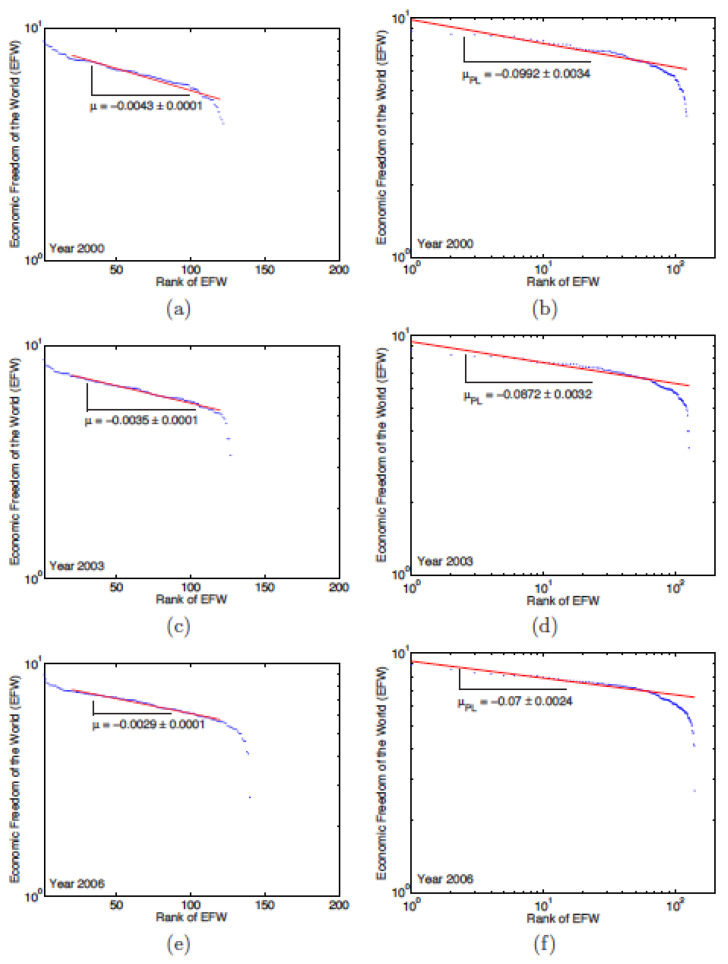
Examples of semi-log [(**a**,**c**,**e**)] and log–log [(**b**,**d**,**f**)] plots of the rank-size relation between the Economic Freedom of the World (EFW) index and the country rank for the years 2000, 2003, and 2006, respectively: the semi-log plots show that the relationship is exponential for countries of high rank (≥20); the log–log plots point to a behaviour close to a power law.

Figure 5a,c,e shows that the EFW has an exponential behaviour for countries with a rank higher than 20. The value of the exponent decreases a little bit more each year and ends up to stabilise at ≃−0.0049 in 2005 and 2006 (see Table 7). The low error bars (less than 0.0001) and the high value regression coefficient (the regression coefficient is greater than 93%) confirm that the data perfectly follow the exponential law.

Figure 5b,d,f shows the power-law behaviour of the EFW. Table 8 reports the values of the exponent of the power law for the 6 studied years. It does not vary much between 2001 and 2004; it falls to −0.0743 in 2005 and −0.007 in 2006. Here again, the effectiveness of the regressions is high, between ∼89% and 93%. This indicates that the data follows a power law.

**Figure 6:**
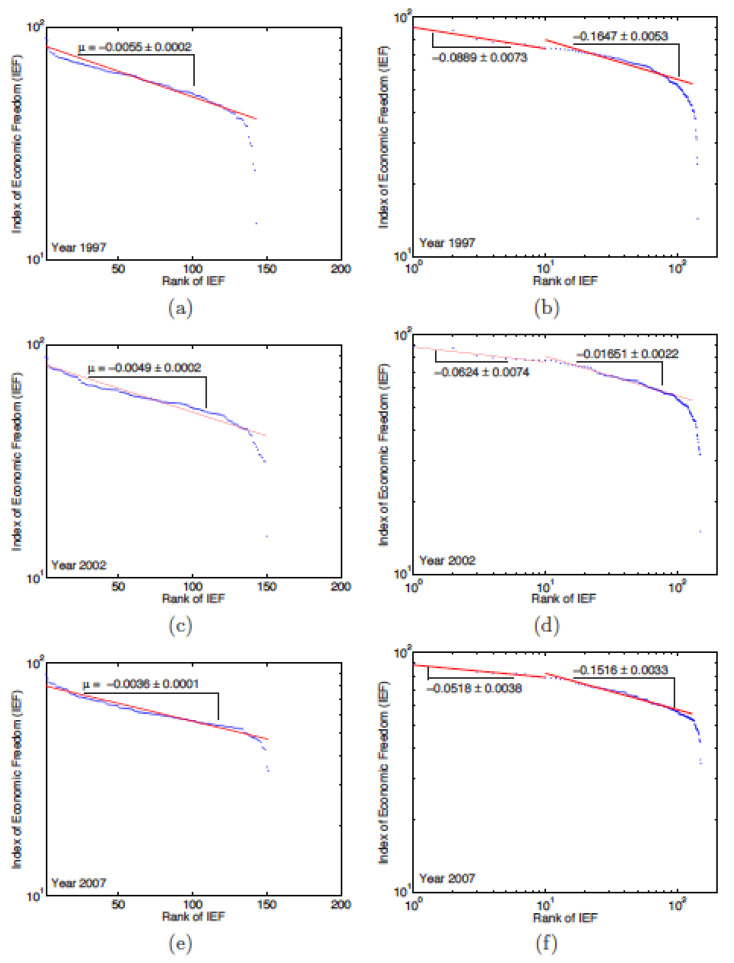
Examples of semi-log [(**a**,**c**,**e**)] and log–log [(**b**,**d**,**f**)] plots of the rank-size relation between the Index of Economic Freedom (IEF) and the country rank for the years 1997, 2002 and, 2007, respectively; the semi-log plots show that the IEF ranking has an exponential behaviour; the log–log plots point to the existence of a transitional point between two different power laws, i.e., near rank 10.

For the IEF, the semi-log graphs, see Figure 6a,c,e, indicates an exponential behavior according to the rank of countries. The exponent decreases every year, going down from −0.006 in 1996 to −0.0036 in 2007 (see Table 9). The regression coefficient shows that the exponential law has been “perfectly” followed since 2003, a year for which the efficiency of the regression exceeds 90%.

Unlike the EFW index, for which the data follow a power law for all ranks, Figure 6b,d,f shows a transition point between 2 different power law for the IEF, near rank 10. The exponent of the law for countries with a rank below 10 “increases“ over the years, from −0.0931 in 1996 to −0.0518 in 2007. The exponent for countries with rank higher than 10 remains relatively stable ≃−0.016 over the 12 years here studied (see Table 10).

It should be noted that countries with low EF (those which have a very high rank) follow neither a power law nor an exponential law; this feature holds for both indices. The difficulty of performing economic measures for these countries can explain that the index values are fraught with errors that are not possible to compensate. These countries are often those with a minimally developed economy, weakly connected to their outside world, apparently subject to the will of a dictator.

### 3.6. Comparison of both Indices

The purpose of this section is to compare the indices, whence it is necessary to restrict the observation “period“ at the largest but common year interval. We should also take into account the countries common to both sets. That leaves 138 countries to be examined over a period extending from 2000 to 2006, i.e., 2 sets of 862 data points.

To have a meaningful comparison, it is best to “normalise“ the index values in an observation interval; here we choose the interval to be [0, 1]. To do so, it is sufficient to divide the values of the EFW index by 10 and those of the IEF by 100.

**Figure 7:**
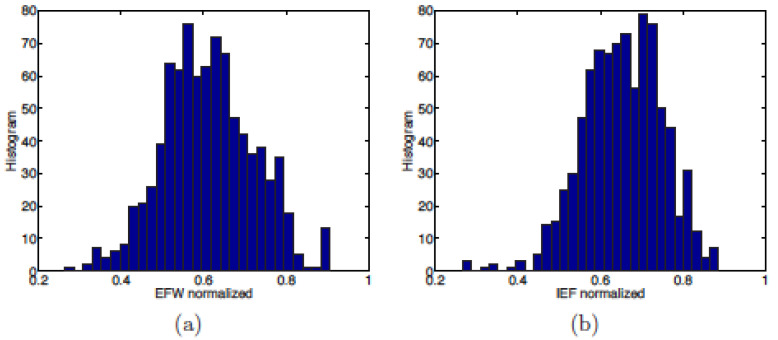
Histogram of (**a**) Economic Freedom of the World (EFW) and (**b**) Index of Economic Freedom (IEF) values for the 862 data points, common to both indices, normalised over [0, 1].

The distributions of the 862 data points are reproduced in Figure 7 for both indices. The average of the EFW values is ≃0.6542, while the average for the IEF is slightly lower at ≃0.6118. This shows that the EFW gives, on average, an index value slightly greater than that given by the IEF for the same country (see below in Table 11).

**Figure 8:**
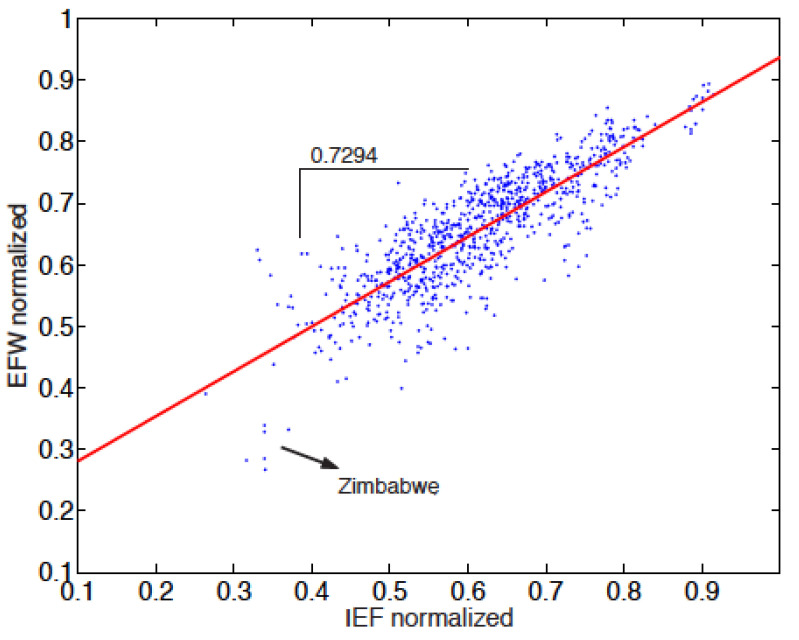
Scatter plot of the relationship between the Economic Freedom of the World (EFW) index and the Index of Economic Freedom (IEF) normalised values. The regression slope points to a linear relationship of ∼0.7294. This value, statistically significant, lower than 1, confirms that the IEF is “more conservative“ than the EFW index. The worst EFW country (Zimbabwe) position is emphasised for framing of the data.

In order to confirm that the IEF is more conservative than the EFW index, it is interesting to represent the EFW values according to the IEF values. This is done in Figure 8. By calculating the linear regression coefficient, the slope is found to be 0.7294. This value is markedly less than 1, whence confirming that the EFW gives index values greater than the IEF for a given country.

## 4. Relationship between Economic Freedom and Wealth of Countries

As recalled here above, many studies show a strong relationship between economic freedom and the wealth of a country, i.e., between EF and the country gross domestic product (GDP). In this section, the goal is to evidence this relationship.

**Figure 9:**
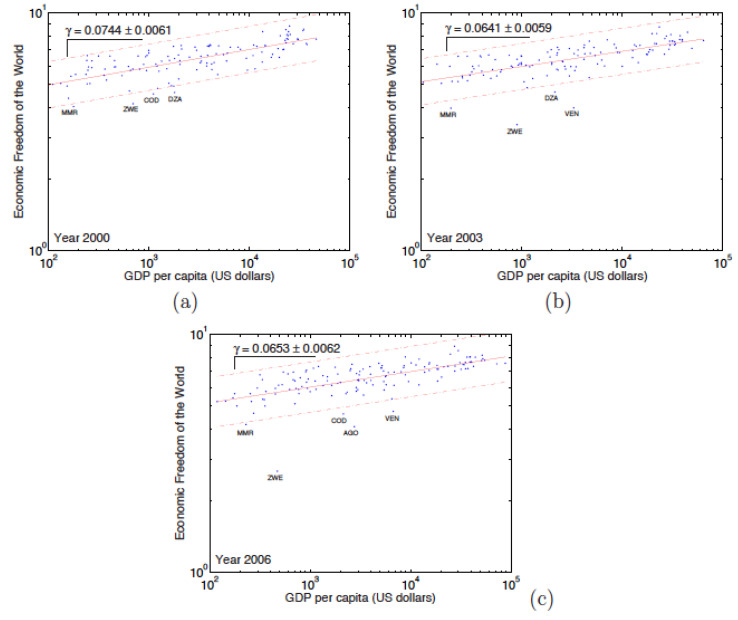
Examples of log–log plot of the Economic Freedom of the World (EFW) Index with respect to country’s gross domestic product (GDP) for the years (**a**) 2000, (**b**) 2003, and (**c**) 2006. This relationship is characterised by a power law, with an exponent γ≃0.674. The dotted lines encompass the region for which the data is within twice the standard deviation away from the trend.

**Figure 10:**
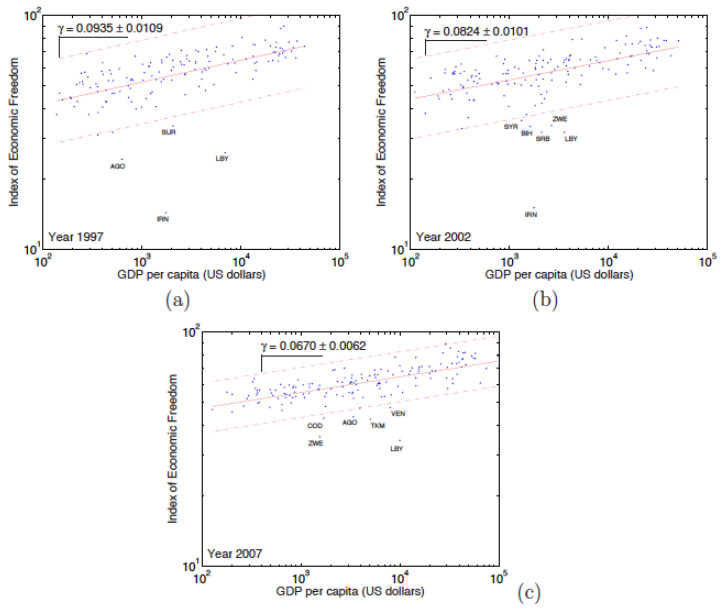
Examples of log–log plots of the Index of Economic Freedom (IEF) relationship to the country’s gross domestic product (GDP) for the years (**a**) 1997, (**b**) 2002, and (**c**) 2007. This relationship is characterised by an evolutive power law. The dotted lines limit the region for which the data are located within a maximum distance equal to twice the standard deviation; the few outliers have been removed for calculating the power law exponent γ.

A graphic representation of EF according to the GDP, on Figure 9 and Figure 10, shows that the relationship translates into a power law, i.e., thereby defining the exponent γ,

(4) $$INDEX≃GDP^{\gamma }\;.$$

A positive exponent (γ>0) indicates a “positive relationship“ between EF and the GDP. This would mean that the freest countries are the richest ones. A negative exponent indicates a negative correlation: the freest countries would be the less rich ones.

The existence of this law is very important from an economic point of view. Indeed, it allows us to know the wealth which a country should have as a function of its level of economic freedom. By estimating the influence that a government decision will have on the economic freedom index of that country, it is possible to directly measure the impact of a government policy on the economy of the country. Moreover, the existence of this (simple) law will enable countries to be classified according to their position on the power law. Countries that are located above the law are countries that have a lower gross domestic product than they should for their level of economic freedom. These countries can be said to be ‘underperforming’.

On the other hand, the countries that are located below the law are countries that have a gross domestic product greater than that which it should have. These countries are ‘over-performing’.

On Table 9, we report the exponential law parameter (λ) between the IEF and the rank (*r*) of the IEF, the Standard Error (Δλ) and its Relative Error (Δλ/λ), together with the efficiency of the regression ($R^{2}$). The λ value decreases each year (in absolute value); it increases from −0.006 in 1996 to −0.0036 in 2007. The efficiency of the regression shows that the data follow an exponential law, rather perfectly since 2003, when the efficiency of the regression exceeds 90%.

On Table 10, we report the (Zipf) rank-size law exponent (ν) between the IEF and the rank (*r*) of IEF, the Standard Error (Δν), the Relative Standard Error (Δν/ν), and the yearly regression coefficients ($R^{2}$), for the observed different regimes. While the exponent for countries of rank below 10 decreases over the years the exponent for countries of rank higher than 10 remains relatively stable, near the value −0.016 over the 12 years of the study.

On Table 11, we report the main characteristics (average and standard deviation) of the normalised EFW and IEF data for the 138 countries out of the 7 years (i.e., 862 data points). The EFW mean is slightly higher than that for the EFW data. The coefficient of variation (σ/μ) shows a weak dispersion in both cases.

On Table 12, we list countries (The ISO 3166-1 code is used to facilitate the presentation of data) for which the EFW Index does not comply with the power law, i.e., the data points are located outside the area limited by twice the standard deviation from the power law.

Figure 9 and Figure 10 clearly show that all countries, with a few exceptions obey the power law. The variation coefficient (σ/μ) shows a weak dispersion on both cases, because the countries are almost all in an interval corresponding to twice the standard deviation. For the EFW, the countries that pose a problem are Algeria, the Republic of Congo, Burma, and Zimbabwe, but also Venezuela since 2002. As regards the IEF, the problematic countries are more numerous: among these are Angola, Bosnia, Iran, Laos, Libya, and Zimbabwe. Venezuela is only an IEF problem since 2004. The lists of such countries are included in Table 12 and Table 13 for each year of interest. In Table 12, we report the list of countries *i* for which the EFW Index values do not comply with the power law. In Table 13, we report the list of countries *i* for which the IEF Index does not comply with the power law.

The exponent γ values for the period 2000 to 2006 relationship between EFW and GDP are reported in Table 14, while the γ values for the IEF for the 1996 to 2007 period are shown in Table 15. In the case of the EFW (see Table 14), the exponent of the law in question remains stable on the 7 years with an average value ≃ 0.0674. Notice that the regressions coefficients for the EFW–GDP relation are not as high as in the case of the exponential and power (rank-size) laws. For the IEF, there are 3 periods on the 12 years during which the exponent holds different behaviours. For the 1996 to 2000 years, the exponent has an average value equal to 0.0948, which remains stable around this value over these 5 years. The second phase, which extends over the years 2001 to 2005, is a transition period during which the value of the exponent falls down. It ends up to some stabilisation around 0.0666 during the third period (2006–2007). The efficiency of the regressions is not very good, except for the third period during which $R^{2}$ is approaching 50%. Therefore, it may be conjectured that the IEF corrections, added in 2006, are bearing fruit.

In Table 15, we report the power law exponent (γ) between the IEF and GDP, the standard error (Δγ), the relative error (Δγ/γ), and the ($R^{2}$) regression coefficient. There are 3 periods to be noticed in which the exponent adopts different behaviours. For the years 1996 to 2000, the exponent has an average value ≃0.0948 and remains stable for about 5 years. The second phase, which is spread over the years 2001 to 2005, is a transitional period during which the value of the exponent falls down. It ends up stabilising around 0.0666 on the third and latest period (2006–2007). Notice that the regression coefficient is not very high: $R^{2}∼0.376$.

## 5. Conclusions

Let us recall the research questions: can one find an empirical law for describing the economic freedom (EF) of nations through the main measure indices, i.e., the Economic Freedom of the World (EFW) index [38] and the Index of Economic Freedom (IEF) [39]? What simple empirical laws can be found through a simple analysis of rank-size laws? Are such laws of interest for discussing the main determinant, according to the literature, i.e., each country’s GDP?

We have taken some data pertaining to the 1997–2007 period, that is before 2008, thus before a recent “financial crisis”, in order not to involve “multiple exaggerated developments” [43], but nevertheless in order to include a drastic turning point, 11 September 2001, following another geo-economico-political event, the fall of the Berlin wall. We have pointed out that the study of EF should develop over two distinct periods, at this time, mainly because the index’s 2008 definition of economic freedom has been modified. In so doing we have selected data, leading to 138 countries examined over a period extending from 2000 to 2006, thus 2 sets of 862 data points.

We have found that the rank distributions obey either an exponential or a power law or a mixed behaviour. The EFW rank relationship is exponential for countries of high rank (≥20), but log-log plots point to a behaviour close to a power law when considering the whole sample. In contrast, the IEF overall ranking has an exponential behaviour. Interestingly, IEF rank-size rule log-log plots point to the existence of a transitional point between two different power laws, i.e., near rank 10.

Besides, the IEF appears to be “more conservative” than the EFW index.

Moreover, when searching for (analytical law) correlations between the country GDP and either EF indices (we have not looked for regressions between these macroeconomic variables and the various “pillars” of the indices, the literature is already abundant), we have distinguished regional aspects, i.e., defining six continents. We find that the EFW index relationship to country GDP is characterised by a power law, with a rather stable exponent (γ≃0.674) as a function of time. In contrast, the IEF relationship with the country’s gross domestic product points to a downward evolutive power law parameter as a function of time. Markedly the two studied indices provide different aspects of EF.

In so doing, we add numerical considerations to the literature, as should be somewhat expected by econophysics research, for this special issue, but presenting to others a different perspective. The rank-size law approach seems original for the present topics. It brings some information on the “statistical universality” of EF during the considered time interval. Thus we expect to open gates for rigorous approaches, i.e., stressing objectiveness in the modelling, rather than ideological bases.

Thereafter, suggestions for further research can be listed: among others, one could consider other time intervals; for example including the 2008 financial crisis, and nowadays considering the COVID-19 pandemic. This is left for our expected paper II. On the other hand, It would be nice to have more “economic considerations” and “historical considerations”, looking at each pillar separately in more detail. For example, one could consider some renormalisation of the indices, taking into account, size (and type) of governments, size of country populations, inflation rates, foreign direct investments, health burden, etc., on one hand, and on the other hand, migration factors, religious factors, education levels, trade union strengths, pandemic constraints, local climate, etc., all of which presents quite a numerical challenge to econophysicists.

## Figures and Tables

**Table 1:** Summary of (rounded) main statistical characteristics of the economic freedom indicators distributions, i.e., the Economic Freedom of the World (EFW) index and Index of Economic Freedom (IEF), according to the examined time interval ΔT for the number *N* of data points.

	ΔT (yrs)	*N*	Mean (μ)	St.Dev. (σ)	Var. ($\sigma^{2}$)	CoV. (σ/μ)	Skewn.	Kurt.
EFW	7	908	6.49	0.98	0.96	0.151	−0.3567	3.3670
IEF	12	1784	58.79	11.97	143.34	0.2036	−0.2373	3.5416

**Table 2:** Kolmogorov–Smirnov (KS) test for the adjustment of data from EFW and IEF to a normal distribution. The distances of KS (DKS) and the *p*-values indicate that KS tests are statistically significant. It is therefore allowed to conclude that the EFW and IEF values follow a normal law, with μ= 6.49 and 58.79 and variance $\sigma^{2}$ = 0.96 and 143.34, respectively.

Kolmogorov–Smirnov (KS) Test	EFW	IEF
*p*-value	0.1088	0.0633
Gaussian Distribution Critical Value	0.0449	0.0321
Significance Level	0.05	0.05
Number of data points	908	1784
DKS	0.0399	0.0310

**Table 3:** 2006 Economic Freedom of the World (EFW) Index values for the 20 freest countries.

2006 EFW Ranking					
Rank	Country	2006	Rank	Country	2006
1	Hong-Kong	8.94	11	Estonia	7.89
2	Singapore	8.57	12	Iceland	7.8
3	New Zealand	8.28	13	Denmark	7.78
4	Switzerland	8.20	14	Finland	7.69
5	United Kingdom	8.07	15	Austria	7.66
6	Chile	8.06	16	Netherlands	7.65
7	Canada	8.05	17	Germany	7.64
8	Australia	8.04	18	Taiwan	7.63
8	United States	8.04	19	Kuwait	7.62
10	Ireland	7.92	20	Slovak Rep.	7.61

**Table 4:** 2006 Economic Freedom of the World (EFW) Index values for the 21 least free countries. Unlike the 20 freest countries on the planet, the 21 least free countries are almost all in Africa (16 of the 21).

2006 EFW Ranking					
Rank	Country	2006	Rank	Country	2006
121	Ethiopia	5.64	131	Burundi	5.23
121	Ukraine	5.64	131	Rwanda	5.23
123	Burkina Faso	5.63	133	Chad	5.12
124	Algeria	5.57	134	Central Africa Rep.	5.01
125	Syria	5.54	134	Guinea-Bissau	5.01
126	Malawi	5.42	136	Venezuela	4.76
127	Gabon	5.37	137	Niger	4.67
128	Nepal	5.35	138	Congo, Rep. of	4.64
129	Togo	5.33	139	Myanmar	4.19
130	Congo, Dem. Rep.	5.25	140	Angola	4.10
			141	Zimbabwe	2.67

**Table 5:** 2006 Index of Economic Freedom (IEF) values for the 20 freest countries.

2006 IEF Ranking					
Rank	Country	2006	Rank	Country	2006
1	Hong-Kong	88.6	11	Iceland	75.8
2	Singapore	88.0	12	Denmark	75.4
3	Ireland	82.2	12	Netherlands, The	75.4
4	New Zealand	82.0	14	Luxembourg	75.3
5	United States	81.2	15	Estonia	74.9
6	United Kingdom	80.4	16	Japan	73.3
7	Australia	79.9	17	Finland	72.9
8	Switzerland	78.9	18	Bahamas, The	72.3
9	Chile	78.0	19	Barbados	71.9
10	Canada	77.4	20	Cyprus	71.8

**Table 6:** 2006 Index of Economic Freedom (IEF) values for the 20 least free countries.

2006 IEF Ranking					
Rank	Country	2006	Rank	Country	2006
138	Chad	50.0	148	Iran	45.0
139	Haiti	49.2	149	Venezuela	44.6
140	Nigeria	48.7	150	Turkmenistan	43.8
140	Burundi	48.7	150	Congo. Rep. of	43.8
140	Uzbekistan	48.7	152	Angola	43.5
143	Laos	47.5	153	Burma	40.0
143	Belarus	47.5	154	Zimbabwe	33.5
145	Togo	47.3	155	Libya	33.2
146	Guinea-Bissau	46.5	156	Cuba	29.3
147	Sierra Leone	45.2	157	Korea. North	4.0

**Table 7:** Yearly evolution of the λ exponent in the assumed empirical exponential law between the EFW index and the rank (*r*), the standard error (Δλ), its relative value (Δλ/λ), and the efficiency ($R^{2}$) of the regression. The low error bar values (less than 0.0001) and the effectiveness of the regressions (≥93%) confirm that the data are perfectly following the exponential law.

	EFW $∼e^{\lambda r}$			
Year	λ	Δλ	Δλ/λ	$R^{2}$
2000	−0.0043	0.0001	0.0272	0.9316
2001	−0.0039	0.0001	0.0257	0.9388
2002	−0.0037	0.0001	0.0208	0.9591
2003	−0.0035	0.0001	0.0129	0.9839
2004	−0.0035	0.0001	0.0068	0.9954
2005	−0.0029	0.0001	0.0107	0.9889
2006	−0.0029	0.0001	0.0113	0.9876

**Table 8:** Yearly evolution of the ν exponent in the empirical power law between the EFW and the rank (*r*), the standard error (Δν), its relative value (Δν/ν), and the efficiency ($R^{2}$) of the regression. The low error bar values (Δν/ν≃3%) and the effectiveness of the regressions confirm that the data is well following a power law.

	EFW $∼r^{\nu }$			
Year	ν	Δν	Δν/ν	$R^{2}$
2000	−0.0992	0.0034	0.0343	0.9161
2001	−0.0907	0.0029	0.0314	0.9285
2002	−0.0890	0.0029	0.0328	0.9226
2003	−0.0872	0.0032	0.0369	0.9038
2004	−0.0857	0.0034	0.0393	0.8924
2005	−0.0743	0.0023	0.0306	0.9319
2006	−0.0700	0.0024	0.0344	0.9154

**Table 9:** Yearly evolution of the λ exponent in the empirical exponential law between the IEF and the rank (*r*), the standard error (Δλ), its relative error (Δλ/λ), and the efficiency ($R^{2}$) of the regression. The low error bar values (Δλ/λ≃2 to 4%) and the effectiveness of the regressions confirm that the data are closely following a power law.

	IEF $∼e^{\lambda r}$			
**Year**	λ	Δλ	Δλ/λ	$R^{2}$
1996	−0.0060	0.0003	0.0422	0.8087
1997	−0.0055	0.0002	0.0405	0.8124
1998	−0.0057	0.0002	0.0385	0.8211
1999	−0.0054	0.0002	0.0416	0.7919
2000	−0.0051	0.0002	0.0382	0.8185
2001	−0.0050	0.0002	0.0345	0.8508
2002	−0.0049	0.0002	0.0381	0.8235
2003	−0.0044	0.0001	0.0212	0.9374
2004	−0.0043	0.0001	0.0241	0.9215
2005	−0.0041	0.0001	0.0246	0.9180
2006	−0.0037	0.0001	0.0238	0.9223
2007	−0.0036	0.0001	0.0227	0.9285

**Table 10:** Yearly evolution of the Zipf law exponent (ν) between the IEF and the rank (*r*) of IEF, the Standard Error (Δν), the Relative Standard Error (Δν/ν), and the Regression Coefficient ($R^{2}$). While the exponent for countries of rank below 10 decreases over the years, the exponent for countries of rank higher than 10 remains relatively stable, near the value −0.016 over the 12 years of the study.

	IEF $∼r^{\nu }$							
	r≤10				r∈[10−100]			
Year	ν	Δν	Δν/ν (%)	$R^{2}$ (%)	ν	Δν	Δν/ν (%)	$R^{2}$ (%)
1996	−0.0931	0.0071	7.60	95.58	−0.1820	0.0056	3.09	92.16
1997	−0.0889	0.0073	8.26	94.82	−0.1647	0.0053	3.25	91.43
1998	−0.0808	0.0099	12.28	89.24	−0.1505	0.0044	2.92	92.96
1999	−0.0797	0.0079	9.90	92.73	−0.1477	0.0029	1.95	96.73
2000	−0.0807	0.0089	10.97	91.21	−0.1504	0.0030	1.98	96.63
2001	−0.0723	0.0058	8.02	95.11	−0.1634	0.0042	2.54	94.57
2002	−0.0624	0.0074	11.80	89.97	−0.1651	0.0022	1.36	98.38
2003	−0.0686	0.0070	10.17	92.35	−0.1704	0.0031	1.81	97.18
2004	−0.0690	0.0092	13.35	87.52	−0.1690	0.0024	1.45	98.16
2005	−0.0717	0.0095	13.26	87.67	−0.1678	0.0024	1.40	98.28
2006	−0.0564	0.0047	8.29	94.78	−0.1522	0.0022	1.45	98.17
2007	−0.0518	0.0038	7.33	95.88	−0.1516	0.0022	1.47	98.11

**Table 11:** Summary of (rounded) main statistical characteristics for the so called “normalized” EFW and IEF distributions of the economic freedom indicators, according to the number of countries $N_{c}$, the examined time interval ΔT, whence the number *N* of data points.

Variable	$N_{c}$	ΔT (Years)	*N*	Mean (μ)	StDev (σ)	CoV (σ/μ)
EFW	138	7	862	0.6542	0.0948	0.1449
IEF	138	7	862	0.6118	0.1094	0.1788

**Table 12:** List of countries for which the EFW Index does not comply with the power law, i.e., are located outside the area limited by twice the standard deviation from the power law.

EFW	
Year	Countries
2000	DZA-COD-MMR-ZWE
2001	DZA-ZWE
2002	DZA-COD-MMR-VEN-ZWE
2003	DZA-MMR-VEN-ZWE
2004	DZA-COD-VEN-ZWE
2005	DZA-COD-VEN-ZWE
2006	AGO-COD-MMR-VEN-ZWE

**Table 13:** List of countries fo which the IEF does not comply with the power law, i.e., are located outside the area limited by twice the standard deviation from the power law.

IEF	
Year	Countries
1996	AGO-AZE-IRN-LBY
1997	AGO-IRN-LBY-SUR
1998	AGO-BIH-IRN-LOA-LBY-UZB
1999	AGO-BIH-COG-IRN-LAO-LBY-UZB
2000	AGO-COG-IRN-LOA-LBY
2001	BLR-BIH-LOA-LBY
2002	BIH-IRN-LBY-SRB-SYR-ZWE
2003	BLR-BIH-LBY–SYR-ZWE
2004	BLR-LBY-SYR-VEN-ZWE
2005	LBY-VEN-ZWE
2006	AGO-COD-LBY-TKM-VEN-ZWE
2007	AGO-COD-LBY-TKM-VEN-ZWE

**Table 14:** Yearly evolution of the power law exponent (γ) between the EFW and GDP, the standard error (Δγ), the relative error bar (Δγ/γ) and the efficiency ($R^{2}$) of the regression. The power law exponent remains rather stable over the 7 years with an average value ≃ 0.0674 (±0.004).

	EFW ∼ ${\mathrm{GDP}}^{\gamma }$			
Year	γ	Δγ	Δγ/γ	$R^{2}$
2000	0.0744	0.0061	0.0824	0.5490
2001	0.0669	0.0061	0.0917	0.4959
2002	0.0636	0.0062	0.0978	0.4636
2003	0.0641	0.0059	0.0922	0.4847
2004	0.0705	0.0057	0.0814	0.5410
2005	0.0667	0.0062	0.0934	0.4540
2006	0.0653	0.0062	0.0952	0.4443

**Table 15:** Yearly evolution of the power law exponent (γ) between the IEF and GDP, the standard error (Δγ), the relative error (Δγ/γ), and the efficiency ($R^{2}$) of the regression. There are 3 periods to be noticed in which the exponent adopts different behaviours. For the years 1996 to 2000, the exponent has an average value ≃0.0948 and remains stable (≃0.09) for about 5 years. The second phase spreads over the years 2001 to 2005, is a transitional period during which the value of the exponent falls down. It ends up stabilising around 0.0666 on the third and latest period (2006–2007). Notice that the regression coefficient ($R^{2}$) is not very high.

	IEF ∼ ${\mathrm{GDP}}^{\gamma }$			
Year	γ	Δγ	Δγ/γ	$R^{2}$
1996	0.0940	0.0117	0.1248	0.3255
1997	0.0935	0.0109	0.1163	0.3439
1998	0.0994	0.0113	0.1140	0.3435
1999	0.0956	0.0112	0.1166	0.3261
2000	0.0915	0.0099	0.1086	0.3583
2001	0.0870	0.0098	0.1131	0.3472
2002	0.0824	0.0101	0.1224	0.3107
2003	0.0802	0.0075	0.0940	0.4332
2004	0.0773	0.0073	0.0947	0.4313
2005	0.0728	0.0070	0.0956	0.4267
2006	0.0662	0.0064	0.0961	0.4208
2007	0.0670	0.0062	0.0922	0.4414

## Data Availability

Data sources are mentioned in the text and references; they are freely accessible.
